# Economic Impact of Postoperative Urinary Retention in the US Hospital Setting

**DOI:** 10.36469/001c.121641

**Published:** 2024-08-08

**Authors:** Weijia Wang, Arielle Marks-Anglin, Vladimir Turzhitsky, Robert J. Mark, Aurelio Otero Rosales, Nathaniel W. Bailey, Yiling Jiang, Joseph Abueg, Ira S. Hofer, Toby N. Weingarten

**Affiliations:** 1 Outcomes Research, Merck & Co., Inc., Rahway, New Jersey, USA; 2 Global Medical Affairs, Merck & Co., Inc., Rahway, New Jersey, USA; 3 Global Scientific Affairs, Merck & Co., Inc., Rahway, New Jersey, USA; 4 Biostatistics and Research Decision Sciences Health Economics and Decision Science, Merck Sharp & Dohme (UK) Ltd., London, UK; 5 Department of Anesthesiology, Pain, and Perioperative Medicine; Department of Medicine, Division of Data Driven Medicine Charles Bronfman Institute of Personalized Medicine, Icahn School of Medicine at Mount Sinai, New York, New York, USA; 6 Department of Anesthesiology and Perioperative Medicine Mayo Clinic College of Medicine, Rochester, Minnesota, USA

**Keywords:** postoperative urinary retention, hospital settings, hospital charges, real-world evidence

## Abstract

**Background:** Postoperative urinary retention (POUR) is a common and distressing surgical complication that may be associated with the pharmacological reversal technique of neuromuscular blockade (NMB).

**Objective:** This study aimed to investigate the impact that POUR has on medical charges.

**Methods:** This was a retrospective observational study of adult patients undergoing select surgeries who were administered neuromuscular blockade agent (NMBA), which was pharmacologically reversed between February 2017 and November 2021 using data from the PINC-AI™ Healthcare Database. Patients were divided into 2 groups: those experiencing POUR (composite of retention of urine, insertion of temporary indwelling bladder catheter, insertion of non-indwelling bladder catheter) during index hospitalization following surgery and those without POUR. Surgeries in inpatient and outpatient settings were analyzed separately. A cross-sectional comparison was performed to report total hospital charges for the 2 groups. Furthermore, patients experiencing subsequent POUR events within three days after discharge from index hospitalization were studied.

**Results:** A total of 330 838 inpatients and 437 063 outpatients were included. POUR developed in 13 020 inpatients and 2756 outpatients. Unadjusted results showed that POUR was associated with greater charges in both inpatient (92 529withPOURvs78 556 without POUR, *p* < .001) and outpatient (48 996withPOURvs35 433 without POUR, *p* < .001) settings. After adjusting for confounders, POUR was found to be associated with greater charges with an overall mean adjusted difference of 10 668(9595 760-11 760,p < .001)ininpatientand13 160 (95% CI 11 750−14  571, *p* < .001) in outpatient settings. Charges associated with subsequent POUR events following discharge ranged from 9418inpatientchargesto1694 outpatient charges.

**Conclusions:** Surgical patients who were pharmacologically reversed for NMB and developed a POUR event incurred greater charges than patients without POUR. These findings support the use of NMB reversal agents associated with a lower incidence of POUR.

## BACKGROUND

Postoperative urinary retention (POUR) is a common postoperative complication with a reported incidence between 5% and 75% of patients, with incidence rates varying based on patient, surgical, and anesthetic variables.^1^ Treatment of POUR may require placement of a catheter into the bladder, which can increase the risk for urinary tract infection and other complications.^2^ In addition, patients who experience POUR have been found to have increased duration of post-anesthesia care unit stay and hospital length of stay, and have increased risk of readmission.^3^ Thus, POUR may represent substantial burden and potentially avoidable impact on healthcare resource utilization and economics^1,4,5^; however, there is a lack of detailed evidence regarding its economic impact.

We recently conducted a large retrospective observational study of patients who underwent surgery that required the administration of neuromuscular blocking agents (NMBA) to evaluate for associations between 2 NMB pharmacological reversal techniques and the risk of POUR. The current study provides a unique opportunity to examine the economic impact that POUR complications have for patients undergoing inpatient and ambulatory surgery. Our aim was to study patients with vs without a POUR event to compare the hospital charges incurred by these patient populations to evaluate the hypothesis that POUR will add substantial economic cost for surgical procedures. Medical charges from postdischarge POUR-related follow-up care were also investigated.

## METHODS

### Study Design and Data Source

A retrospective observational study of adult patients undergoing select surgeries who were administered NMBA and pharmacologically reversed was conducted using Premier Inc.’s PINC-AI™ Healthcare Database (PHD). A cross-sectional comparison of 2 inpatient and outpatient cohorts, among those with and without POUR (**Supplementary Table S5**) after the surgeries, was performed to estimate hospital charges associated with having a POUR event at index hospitalization. Furthermore, patient cohorts with at least 1 POUR event within 3 days after discharge from the index hospitalization were studied to describe charges of subsequent POUR events following discharge.

The PHD is a large, comprehensive US hospital-based, service-level, all-payer electronic healthcare database.^6^ It contains information on inpatient discharges, primarily from geographically diverse nonprofit, nongovernmental, community or teaching hospitals and health systems from rural or urban areas.^6^ Hospitals/healthcare systems submit administrative, healthcare utilization, and financial data from patient encounters. With over 108 million inpatient admissions included, approximately 25% of annual US inpatient admissions are represented. The database uses a unique masked identifier to track patients in the same hospital across the inpatient and hospital-based outpatient settings, with the ability to assess hospital length of stay and readmissions to the same hospital. As this study involved analysis of pre-existing, de-identified data, it did not require institutional review board review per US Federal Regulations for the Protection of Human Research Subjects (45 CFR §46). This study adheres to the applicable STROBE guidelines.

### Inclusion and Exclusion Criteria

Surgical encounters occurring between February 1, 2017, and November 30, 2021, were identified. The 4 surgery types included were knee-hip arthroplasty, hernia surgery (inguinal, abdominal), rectal surgery, and laparoscopic surgery. For patients undergoing multiple surgeries during a 30-day calendar period or a given inpatient stay, only the first surgery was included in the analysis. As the database reports only intervention dates with month granularity, surgeries within the same month or prior month were considered as being within the 30-day period. Since the order of inpatient procedures in PHD is unknown, to reduce misclassifying catheterization done prior to the procedure as POUR, only surgery types that do not require catheterization were included (applicable for the index hospitalizations and subsequent POUR events after discharge).

Patients diagnosed with myasthenia gravis or renal failure, or with a history of paralysis, paralyzing stroke, or other neuromuscular disorders, including multiple sclerosis, paraplegia, neuropathies, and neurogenic bladder, were excluded. Patients receiving both sugammadex and neostigmine or diagnosed with COVID-19 were also excluded.

### Outcomes

Patients’ demographic characteristics, treatment patterns, and clinical and economic outcomes were analyzed. Economic outcomes were medical charges related to POUR for index hospitalization and follow-up POUR event. Medical charges for POUR at index hospitalization were defined as the difference between total hospital charges for patients with a POUR event at index hospitalization and for those who did not have a POUR event at index hospitalization. Total hospital charges represent the amount charged or reimbursed for delivering billed goods and services during the hospital encounter. Furthermore, medical charges for subsequent POUR events after discharge were defined as medical charges incurred at inpatient or outpatient setting during the 3-day period after discharge that includes primary or secondary diagnosis of POUR. All charges were adjusted to 2022 U.S. dollars using a Consumer Price Index.^7^

### Statistical Analyses

First, data quality was assessed for POUR events at index hospitalization. Missingness rate, mean, minimum, maximum, and 25th, 50th, and 75th percentiles for charges were reported. Extreme values for charges were removed in a 4-step process, and sensitivity analyses were conducted using remaining records in each step (**Supplementary Tables S1, S2, and S3**).

Next, unadjusted means and 95% confidence intervals (CIs) were reported for medical charges separately for patients with and without a POUR event during index hospitalization, overall and by surgery type. Wilcoxon rank sum tests were used to compare the unadjusted means for the patient groups with or without a POUR event, with *p* values being reported for both the overall cohort and by surgery type.

Then, regression models were developed to estimate adjusted means for charges for the POUR event. First, several univariate generalized linear models (GLMs) were fitted with a selection of potential confounders using the gamma distribution with a log link function to identify significant confounding variables (*p* < .05). These included age (as a numeric continuous variable), gender, race/ethnicity, year of index event, number of comorbidities, institution characteristics (teaching vs non-teaching hospitals) and geographic location. Next, a multivariate GLM was developed with all significant confounding variables using the gamma distribution with a log link function. A modified Park test was performed to confirm whether the gamma distribution was appropriate for this model. Both the coefficient (ƛ) and the 95% interval for the coefficient from the multivariate GLM were reported. Marginal adjusted means and their 95% CIs were estimated for the 2 cohorts overall and by surgery type.

The analysis for estimating medical charges for subsequent POUR events after discharge was limited to patients with at least 1 follow-up POUR event within 3 days of discharge from the index hospitalization. Due to the small sample size, only unadjusted total hospital charges were reported with 95% CI and compared by the Wilcoxon rank sum tests between cohorts. All analyses were performed in SAS 9.4.

## RESULTS

### Study Population

A total of 331 429 inpatients and 437 685 outpatients were identified that met the inclusion and exclusion criteria. After step 1 of the data quality assessments, 330 838 inpatients and 437 063 outpatients were included in the main analyses. Attritions after steps 2 to 4 of the data quality assessment (used for sensitivity analysis) can be found in **Supplementary Tables S1 and S2**.

Demographic characteristics and treatment patterns for inpatient and outpatient settings are shown in **Supplementary Table S4**. A total of 13 020 (3.9%) inpatients had a POUR event, with 53.1% of these patients undergoing knee-hip arthroplasty (n = 6915) and 24.2% of rectal surgery (n = 3151). A total of 2756 (0.6%) outpatients had a POUR event, with 65.9% of these patients undergoing a hernia surgery (n = 1817) and 21.9% a knee-hip arthroplasty (n = 604).

### Hospital Charges for Index Hospitalization

**Index inpatient hospitalization charges:** Unadjusted charges were higher overall at index hospitalization for inpatients with POUR than for inpatients without POUR ($92 529 vs $78 556, *p* < .001; **Table 1**). Results showed that laparoscopic surgery had the highest charges for inpatients with POUR ($114 131 vs $81 083, *p* < .001), followed by rectal surgery (108 553 vs $83 657, *p* < .001).

**Table 1.** Unadjusted Hospital Charges With and Without POUR Following Inpatient Surgery

**Surgery Type**	**Total Hospital Charges**						**Wilcoxon Test *p* Value**
	**POUR**			**No POUR**			
	**n**	**Mean, $ (SD)**	**95% CI**	**n**	**Mean, $ (SD)**	**95% CI**	
Overall	13 020	92 529 (83 777.0)	91 089.8- 93 968.1	317 818	78 556 (57 704.5)	78 355.1- 78 756.3	<.0001
Hernia	1600	91 322 (84 685.1)	87 169.0- 95 474.3	36 881	77 630 (69 834.9)	76 917.0- 78 342.5	<.0001
Knee or hip surgery	6915	81 277 (56 334.2)	79 948.9- 82 604.9	160 681	75 686 (47 203.7)	75 454.9- 75 916.5	<.0001
Laparoscopic surgeries	1354	114 131 (123 527.2)	107 545.1- 120 716.1	45 888	81 083 (68 001.5)	80 460.6- 81 705.0	<.0001
Rectal	3151	108 553 (105 412.0)	104 870.8- 112 234.7	74 368	83 657 (64 000.3)	83 196.6- 84 116.6	<.0001

Abbreviations: CI, confidence interval; POUR, postoperative urinary retention.

Overall adjusted charges were approximately 12% greater for inpatients with POUR than for inpatients without POUR ($100 630 vs $89 962, *p* < .001; **Table 2**), with a mean adjusted difference of $10 668 (95% CI, $9576-$11 760). Differences in charges were greatest for laparoscopic surgeries: $27 611 (95% CI, $25 470-$28 401; *p* < .001), from $18 887 to $26 935. This was followed by rectal surgery: $20 374 (95% CI, $17 968-$22 780; *p* < .001), from $94 907 to $115 281. The smallest difference in charges due to POUR was $3168 (95% CI $1990-$4347; *p* < .001) from $89411 to $92 579 in patients undergoing knee-hip surgery.

**Table 2.** Generalized Linear Model: Adjusted Hospital Charges With and Without POUR Following Inpatient Surgery

**Surgery Type^a^**	**Total Hospital Charges, $**				**Difference in Adjusted Means**	**95% CI for Difference in Means**	**Coefficient *p* Value**
	**POUR**		**No POUR**				
	**Adjusted Mean**	**95% CI**	**Adjusted Mean**	**95% CI**			
Overall^b^	100 630	95 199-106 372	89 962	85 162-95 033	10 668	9576.20-11 760.02	<.0001
Hernia	98 413	92 639-104 546	87 397	82 716-92 342	11 016	8384.61-13 647.44	
Knee or hip	92 579	87 532-97 917	89 411	84 641-94 450	3168	1989.95-4346.63	
Laparoscopic	116 138	109 220-123 495	88 527	83 792-93 530	27 611	24 018.92-31 203.45	
Rectal	115 281	108 819-⁠122 126	94 907	89 838-⁠100 262	20 374	17 968.07-⁠22 779.89	

^a^For adjusted means by surgery type, the model adjusts for age, gender, race, ethnicity, index year, number of comorbidities, whether provider is a teaching hospital, US census region of provider, surgery type, and an interaction between POUR and surgery type.

^b^Overall model adjusts for age, gender, race, ethnicity, index year, number of comorbidities, whether provider is a teaching hospital, US census region of provider, and surgery type.

Abbreviations: CI, confidence interval; POUR, postoperative urinary retention.

**Index outpatient hospitalization charges:** Unadjusted overall charges were higher for patients with an index POUR event than for patients without POUR ($48 996 vs $35 433, *p*<.001; **Table 3**). Knee-hip surgery had the highest hospital charges for outpatients with POUR ($68 118 vs $63 338, *p* < .0001), followed by rectal surgery ($60 811 vs $27 886, *p* < .0001).

**Table 3.** Unadjusted Hospital Charges, With and Without POUR Following Outpatient Surgery

**Surgery Type**	**Total Hospital Charges ($)**						**Wilcoxon Test** ***p* Value**
	**POUR**			**No POUR**			
	**n**	**Mean (SD)**	**95% CI**	**n**	**Mean (SD)**	**95% CI**	
Overall	2756	48 996 (32 623.6)	47 777.6-50 214.6	434 307	35 433 (25 070.7)	35 358.4-35 507.5	<.0001
Hernia	1817	41 238 (26 616.5)	40 013.5-42 462.7	324 295	31 660 (20 428.5)	31 589.2-31 729.8	<.0001
Knee or hip surgery	604	68 118 (37 112.6)	65 152.7-71 084.0	48 631	63 338 (33 934.2)	63 036.6-63 639.8	.0002
Laparoscopic surgeries	216	54 276 (33 488.6)	49 784.5-58 767.0	49 534	34 546 (25 601.7)	34 320.8-34 771.7	<.0001
Rectal	119	60 811 (43 288.2)	52 952.7-⁠68 669.1	11 847	27 886 (22 462.1)	27 481.1-⁠28 290.1	<.0001

Abbreviations: CI, confidence interval; POUR, postoperative urinary retention.

Overall adjusted charges were approximately 27% greater for outpatients with POUR than for outpatients without POUR, with an adjusted mean difference of $13 160 (95% CI, $11 750-$14 571; *p* < .001; **Table 4**), from $48 554 to $61 715. The greatest difference in charges was $34 764 for outpatients undergoing rectal surgery (95% CI, $27 657-$41 872; *p* < .001), from $36 213 to $70 977, followed by laparoscopic surgeries ($23 058 [95% CI, $17 983-$28 133], from $45 545 to $68 603, *p* < .001). The smallest difference was $4875 for outpatients undergoing knee-hip surgery (95% CI, $1241-$8509, *p* < .001), from $78 887 to $83 762.

**Table 4.** Generalized Linear Model: Adjusted Hospital Charges, with and without POUR Following Outpatient Surgery

**Surgery Type^a^**	**Total Hospital Charges, $**				**Difference in Adjusted Means**	**95% CI for Difference in Means**	**Coefficient *p* Value**
	**POUR**		**No POUR**				
	**Adjusted Mean**	**95% CI**	**Adjusted Mean**	**95% CI**			
Overall^b^	61 715	(58 473, 65,136)	48 554	(46 180, 51 050)	13 160	(11 750.3, 14 570.5)	<.0001
Hernia	53 949	(51 021, 57,044)	42 560	(40 482, 44 745)	11 389	(9931.5, 12 846.0)	
Knee or hip surgery	83 762	(78 410, 89,479)	78 887	(75 018, 82 957)	4 875	(1240.6, 8508.5)	
Laparoscopic surgeries	68 603	(62 846, 74 888)	45 545	(43 312, 47 894)	23 058	(17 983.0, 28 132.5)	
Rectal	70 977	(63 641, 79 160)	36 213	(34 414, 38 107)	34 764	(27 657.0, 41 871.6)	

^a^For adjusted means by surgery type, the model adjusts for age, gender, race, ethnicity, index year, number of comorbidities, whether provider is a teaching hospital, US census region of provider, surgery type and an interaction between POUR and surgery type.

^b^Overall model adjusts for age, gender, race, ethnicity, index year, number of comorbidities, whether provider is a teaching hospital, US census region of provider, and surgery type.

Abbreviations: CI, confidence interval; POUR, postoperative urinary retention.

### Hospital Charges for Subsequent POUR Events Following Discharge

**Index inpatients with subsequent POUR event following hospital discharge:** The overall index inpatient group experiencing a subsequent POUR event (n = 35) was further divided into 2 groups (**Table 5**): those with a subsequent POUR event in the outpatient setting (n = 33), and those with a subsequent POUR event in the inpatient setting (n = 2). The mean overall charges for 33 subsequent events in outpatient setting were $2878 (median, $1625; interquartile range [IQR], $1099-$2729). Further, 1 subsequent inpatient POUR event developed after a hernia surgery with a charge of $9418 and 1 after rectal surgery with a charge of $6611.

**Table 5.** Hospital Charges for Index Inpatients and Outpatients Who Developed Subsequent POUR Following Hospital Discharge (Unadjusted for Risk Factors)

**Surgery Type**	**n**	**Mean, $ (SD)**	**Median (IQR)**	**Range**
Index inpatients with outpatient subsequent POUR following hospital discharge				
All	33	2878 (4746.3)	1625 (1099-2729)	29-27 241
Hernia	4	4462 (3731.5)	4424 (1235-7688)	1099-7899
Knee or hip surgery	18	3316 (6113.6)	1847 (1246-3268)	29-27 241
Laparoscopic surgeries	1	3589 (NA)	3589 (3589-3589)	3589-3589
Rectal	10	1383 (905.2)	1173 (855-2433)	59-2729
Index inpatients with inpatient subsequent POUR following hospital discharge				
All	2	8014.1 (1984.7)	NA	6611-9417
Hernia	1	9417.5 (NA)	NA	9417-9417
Rectal	1	6610.7 (NA)	NA	6611-6611
Index outpatients with subsequent POUR following hospital discharge				
All	53	1694 (4092.9)	978 (602-1637)	0-30 117
Hernia	50	1732 (4207.3)	981 (631-1637)	0-30 117
Knee or hip surgery	2	1306 (1443.2)	1306 (285-2326)	285-2326
Rectal	1	565 (NA)	565 (565-565)	565-565

Abbreviations: IQR, interquartile range; NA, not applicable; POUR, postoperative urinary retention.

**Index outpatients with subsequent POUR following hospital discharge:** A total of 53 index outpatients developed subsequent POUR events (**Table 5**) with mean overall charges of $1694 (median, $978; IQR, $602-$1637).

Three patients had multiple subsequent events, 1 index inpatient had 2 subsequent outpatient visits, and 2 index outpatients had 2 subsequent outpatient visits. The charges were summed up per patient as related to the subsequent POUR events following hospital discharge.

### Sensitivity Analyses

Results from the sensitivity analyses for inpatients support results from the main analysis, as patients with POUR at index hospitalization were found always to have higher medical charges than patients without POUR (**Supplementary Table S3**).

## DISCUSSION

Results from this retrospective observational study of adult patients after surgery showed that POUR may lead to substantially higher medical charges in both inpatient and outpatient settings. Average charges from 13 020 inpatients and 2756 outpatients who had POUR events showed an 11% to 14% increase in medical charges compared with similar patients receiving similar surgical procedures not complicated by POUR events. Analyses by surgery type revealed that POUR may incur the most substantial charges following laparoscopic surgery.

A few previous studies have investigated the economic burden of POUR for patients and the healthcare system and reported that higher costs were primarily driven by both increased time to hospital discharge and hospital readmissions for urinary retention.^5^ In an observational study investigating different outcomes associated with POUR, patients with POUR were found to have a greater length of hospital stay (0.24 extra days) and needed additional posthospitalization care (OR, 1.3 [95% CI, 1.25-1.4]) when compared with patients without POUR.^8^ These longer hospital stays and unplanned readmissions have direct implications on hospital charges for healthcare systems and decrease the availability of beds for emergency and elective admissions.^5^ Furthermore, the direct treatment of urinary retention also contributes to the economic burden of POUR. Many patients require an indwelling bladder catheter, which may require ongoing outpatient management in urology and specialty clinics. This entails an increase in the utilization of economic resources, and a requirement for clinical staff that can be limited in availability, adding to the total hospital charges associated with POUR.^5^ Findings from current study contribute to this evidence and quantify the economic burden induced by the increased healthcare resources required to manage POUR complications.

All patients in this cohort received NMBA during surgery, which was pharmacologically reversed with either neostigmine or sugammadex administration. Reversal with neostigmine, an acetylcholinesterase inhibitor, has unwanted muscarinic effects and thus must be co-administered with an anticholinergic agent such as glycopyrrolate.^9^ In contrast, sugammadex is devoid of muscarinic effects and thus does not require anticholinergic agent coadministration.^10^ Anticholinergic drugs have direct activity on the bladder musculature, resulting in relaxation of the detrusor muscle, and this action can interfere with micturition.^11^ Intra-operative administration of glycopyrrolate, a common anticholinergic agent, has been found to increase the risk for POUR following ambulatory general surgery.^12^

Recently, research has suggested that the choice of the pharmacological reversal technique for NMB can impact the incidence of POUR. In a study investigating the records of adult patients undergoing unilateral inguinal herniorrhaphy with NMB, fewer patients receiving sugammadex were found to have POUR (3%) than patients receiving neostigmine/glycopyrrolate (15%).^13^ Results from 1 to 2 propensity score–matched models fitted to evaluate the risk of POUR following inguinal hernia repair also showed that sugammadex had significantly lower risk of POUR compared with anticholinesterase (OR, 0.340; *p* < 0.001 [95% CI, 0.198-0.585]).^14^ Furthermore, results from a randomized controlled trial to evaluate efficacy of sugammadex vs neostigmine reported urinary retention only in the group receiving neostigmine (n = 2).^15^ Clinical findings from the current study have been published previously and indicated that the incidence of POUR was twice as great among patients receiving neostigmine/glycopyrrolate compared with sugammadex (5.0% vs 2.4% inpatients; 0.9% vs 0.4% outpatients; both *p* < .0001).^16^ These findings suggest that use of sugammadex over neostigmine/glycopyrrolate as the NMB reversal agent may reduce the incidence of POUR and its associated charges.

### Limitations

This study had several limitations. Data included only commercial sites within the United States and may not reflect national or global trends. Furthermore, clinical variables that were not available in the data set used may have impacted the likelihood of the outcome of interest (POUR). These may include details of preoperative care (eg, treatment given prophylactically to prevent the outcomes of interest), perioperative care, provider bias, surgeon preference, patient anthropometric data (eg, body mass index, which is largely missing), previous patient clinical events or medical history (eg, history of prostate hypertrophy or surgery), the use of intraoperative or postoperative opioids (including their dosing) and fluid intake/balance. Lastly, there was no matching between patients with and without POUR when comparing POUR medical charges at index hospitalization, potentially reducing the comparability of patient groups. However, all applicable variables that may be used in matching in the multivariable regression models were included. Thus, there is control for the confounding effects, increasing the likelihood of creating comparable groups.

## CONCLUSION

Surgical patients who underwent general anesthesia that included an NMBA and pharmacological reversal who had a POUR event incurred greater charges than patients without a POUR event, regardless of surgery type, demographics, and clinical characteristics. As reversal agents for NMB may influence the incidence of POUR, these findings support utilization of clinical practices, including choice of NMB reversal agents associated with a lower incidence of POUR to minimize the economic burden of surgical patients on the healthcare system.

### Ethics Approval and Consent to Participate

This study analyzed claims from a de-identified large US hospital database. As this study involved analysis of pre-existing, deidentified data, it did not require institutional review board review per United States Federal Regulations for the protection of Human Research Subjects (45CRF46), a US federal law. This manuscript adheres to the applicable STROBE guidelines.

### Data Availability

The data that support the findings of this study are available from Premier Inc.’s PINC-AI™ Healthcare Database (PHD), but restrictions apply to the availability of these data, which were used under license for the current study, and so are not publicly available. Data are, however, available from the authors upon reasonable request and with permission of Premier Inc.

### Disclosures

W.W., A.M.A., V.T., R.M., J.A., A.O.R., N.W.B., and Y.J. are employees of Merck Sharpe & Dohme LLC, a subsidiary of Merck & Co., Inc. Rahway, New Jersey, USA (MSD), and may own stock and/or stock options in MSD. I.S.H. received consulting fees from MSD and is the founder and president of Extrico Health. T.N.W. received consulting fees from MSD, Medtronic, Takeda, and Travena. Adelphi Values PROVE was contracted to provide medical writing support in preparing and editing this manuscript.

## Supplementary Material

Online Supplementary Material
