# Supplementary material for: Economic Impact of Postoperative Urinary Retention in the US Hospital Setting
Source: J Health Econ Outcomes Res. 2024 Aug 8;11(2):29–34. doi: 10.36469/001c.121641 (PMC11392480; doi:10.36469/001c.121641)
Supplement: Online Supplementary Material [file jheor_2024_11_2_121641_240959.pdf]

Journal of Health Economics and Outcomes Research

Online Supplementary Material

Economic Impact of Postoperative Urinary Retention in the US Hospital Setting. *JHEOR*. 2024;11(2):29-34. doi:10.36469/jheor.2024.121641

Table S1: Cohort Data Cleaning Attrition – Inpatient

Table S2: Cohort Data Cleaning Attrition – Outpatient

Table S3: Generalized Linear Model – Adjusted Hospital Charges, With and Without Postoperative Urinary Retention Following Surgery Sensitivity Analysis

Table S4: Clinical Characteristics of Surgical Patients With and Without POUR in Both Outpatient and Inpatient Surgical Settings

Table S5: Post-operative Urinary Retention (POUR) Definition

This supplementary material has been provided by the authors to give readers additional information about their work.

This is an open-access article distributed under the terms of the Creative Commons Attribution 4.0 International License (CCBY-4.0). View this license's legal deed at http://creativecommons.org/licenses/by/4.0 and legal code at http://creativecommons.org/licenses/by/4.0/legalcode for more information.

Table S1: Cohort Data Cleaning Attrition – Inpatient

Criteria	Patient counts (N)	Percentage (%)
Cohort before data cleaning	331,429	100.00
Remove patients where cost > charges (Main analysis)	330,838	99.82
Remove extreme values for difference = charges - cost	329,897	99.72
Remove extreme values and zeros for cost	328,196	99.48
Remove extreme values for charges	327,611	99.82

Table S2: Cohort Data Cleaning Attrition – Outpatient

Criteria	Patient Counts (N)	Percentage (%)
Cohort before data cleaning	437,685	100.00
Remove patients where cost > charges (main analysis)	437,063	99.86
Remove extreme values for difference = charges - cost	437,054	100.00
Remove extreme values and zeros for cost	436,047	99.77
Remove extreme values for charges	435,435	99.86

Table S3: Generalized Linear Model – Adjusted Hospital Charges, With and Without Postoperative Urinary Retention Following Surgery Sensitivity Analysis[1]

	Total Hospital Charges	POUR		No POUR		Difference in adj means	95% CI for difference in means
	Sensitivity Analysis	Adj. Mean, $	95%CI	Adj. Mean, $	95%CI		
Inpatient	Step two[2]	96,997	(91,934, 102,338)	88,191	(83,641, 92,989)	8,806	(7,825.11, 9,785.95)
	Step three[3]	95,651	(90,717, 100,854)	87,506	(83,045, 92,208)	8,145	(7,199.16, 9,090.91)
	Step four[4]	94,711	(89,876, 99,806)	87,007	(82,618, 91,630)	7,704	(6,783.94, 8,623.73)
Outpatient	Step two[2]	61,711	(58,470, 65,131)	48,541	(46,168, 51,035)	13,170	(11,760.05, 14,579.79)
	Step three[3]	61,446	(58,221, 64,851)	48,518	(46,147, 51,010)	12,929	(11,528.36, 14,329.03)
	Step four[4]	61,477	(58,265, 64,865)	48,587	(46,225, 51,070)	12,890	(11,496.91, 14,282.76)

[1] The model adjusts for age, gender, race, ethnicity, index year, number of comorbidities, whether provider is a teaching hospital, US census region of provider, and surgery type.

[2] Analytic sample has been trimmed for extreme differences between charges and costs.

[3] Analytic sample has been trimmed for (1) extreme differences between charges and costs, and (2) extreme costs.

[4] Analytic sample has been trimmed for (1) extreme differences between charges and costs, (2) extreme costs, and (3) extreme charges.

Abbreviations: CI, confidence interval; POUR, postoperative retention.

Table S4: Clinical Characteristics of Surgical Patients With and Without POUR in Both Outpatient and Inpatient Surgical Settings

Characteristics	Outpatient		Inpatient	
	**With POUR N= 2,756**	**Without POUR N=434,307**	**With POUR N=13,020**	**Without POUR N=317,818**
Age, years (SD)	67.1 (13.2)	55.1 (15.6)	68.1 (12.5)	62.1 (13.1)
Male (%)	694 (25.2)	162,932 (37.5)	4,833 (37.1)	192,614 (60.6)
Female (%)	2,062 (74.8)	271,375 (62.5)	8,187 (62.9)	125,204 (39.4)
Benign prostatic hyperplasia (%)	677 (24.6)	12,703 (2.9)	2,967 (22.8)	13,527 (4.3)
NMB (%)				
Rocuronium	2,626 (95.3)	418,709 (96.4)	12,241 (94.0)	301,379 (94.8)
Vecuronium	206 (7.5)	20,537 (4.7)	1,257 (9.7)	25,549 (8.0)
Scopolamine	205 (7.4)	41,540 (9.6)	1,491 (11.5)	48,281 (15.2)
Surgery type (%)				
Knee/hip arthroplasty	604 (21.9)	48,631 (11.2)	6,915 (53.1)	160,681 (50.6)
Hernia surgery	1,817 (65.9)	324,295 (74.7)	1,600 (12.3)	36,881 (11.6)
Rectal surgery	119 (4.3)	11,847 (2.7)	3,151 (24.2)	74,368 (23.4)
Laparoscopic surgery	216 (7.8)	49,534 (11.4)	1,354 (10.4)	45,888 (14.4)

Abbreviations: NMB, neuromuscular blockade; POUR, postoperative urinary retention.

Table S5: Post-operative Urinary Retention (POUR) Definition

The ***primary outcome*** post-operative urinary retention (POUR) is defined as:

POUR Code	Type	Description
R33	ICD-10	Retention of urine
51702	CPT	Insertion of temporary indwelling bladder catheter
51701	CPT	Insertion of non-indwelling bladder catheter
51703	CPT	Insertion of non-indwelling bladder catheter, complicated

Abbreviation: ICD-10, *International Classification of Disease 10th version; CPT: Current Procedural Terminology.*
